# *Rpv10.2*: A Haplotype Variant of Locus *Rpv10* Enables New Combinations for Pyramiding Downy Mildew Resistance Traits in Grapevine

**DOI:** 10.3390/plants13182624

**Published:** 2024-09-20

**Authors:** Tim Höschele, Nagarjun Malagol, Salvador Olivella Bori, Sophia Müllner, Reinhard Töpfer, Jürgen Sturm, Eva Zyprian, Oliver Trapp

**Affiliations:** 1 Staatliche Lehr- und Versuchsanstalt im Wein- und Obstbau Weinsberg (LVWO), Traubenplatz 5, 74189 Weinsberg, Germany; 2 Institute for Grapevine Breeding Geilweilerhof, Julius Kühn Institute (JKI), 76833 Siebeldingen, Germany

**Keywords:** grapevine breeding, *Vitis*, downy mildew, QTL, genotyping, phenotyping

## Abstract

In viticulture, pathogens like the oomycete *Plasmopara viticola*, the causal agent of downy mildew, can cause severe yield loss and require extensive application of plant protection chemicals. Breeders are generating pathogen-resistant varieties exploiting American and Asian wild *Vitis* germplasm as sources of resistance. Several loci mediating resistance to *P. viticola* have been identified in the past but may be overcome by specifically adapted strains of the pathogen. Aiming to find and characterize novel loci, a cross population with *Vitis amurensis* ancestry was investigated searching for resistance-correlated quantitative trait loci (QTL). As a prerequisite, a genetic map was generated by analyzing the 244 F_1_ individuals derived from a cross of the downy mildew susceptible *Vitis vinifera* cultivar ‘Tigvoasa’ and the resistant *V. amurensis* pBC1 breeding line We 90-06-12. This genetic map is based on the information from 627 molecular markers including 56 simple sequence repeats and 571 rhAmpSeq markers. A phenotypic characterization of the progeny showed a clear segregation of the resistance traits in the F_1_ population after an experimental inoculation of leaf discs with downy mildew. Combining genetic and phenotypic data, an analysis for QTL revealed a major locus on linkage Group 9 that correlates strongly with the resistance to downy mildew. The locus was mapped to a region of about 80 kb on the PN40024 (12x.V2) grapevine reference genome. This genomic region co-localizes with the formerly identified locus *Rpv10* from the grapevine cultivar ‘Solaris’. As we found different allele sizes of the locus-linked SSR markers than those characterizing the known *Rpv10* locus and differences in the sequence of a candidate gene, it was regarded as a haplotype variant and named *Rpv10.2*.

## 1. Introduction

To minimize the risk of yield loss or quality reduction culturing crops for human consumption, plant protection strategies were established to safeguard the production in the permanent struggle with pests and diseases [1]. Regarding *Plasmopara viticola* (Berk. & Curt.) Berl. & de Toni, the downy mildew (DM)-causing agent in grapevines, wine growers have to fight the pathogen since it was introduced into Europe in 1878 [2]. As traditional European *Vitis vinifera* (subsp. *vinifera* L.) cultivars are susceptible to *P. viticola*, an intense application of phytochemicals is necessary to prevent yield loss [3,4]. Besides environmental concerns raised by the high amount of fungicides used [5], pathogens may be under selective pressure to develop fungicide-resistant strains [6,7]. As a more environmentally friendly alternative, grapevine breeders aim to develop new genetically resistant cultivars. Currently, 37 different loci linked to downy mildew resistance have been described (www.vivc.de/loci, accessed on 1 August 2024), and three of them are frequently used in breeding, *Rpv3*, as well as *Rpv10* and *Rpv12*. In 2004, a major QTL named *Rpv3* was discovered [8,9]. This locus was introgressed from a North American wild ancestor to the *V. vinifera* cultivar ‘Regent’ [10,11]. North American wild species like *Vitis rupestris* (Scheele) or *Vitis riparia* (Michx.) are especially thought to have undergone a coevolution with the endemic oomycete *P. viticola* [2,12,13], resulting in resistant accessions. Nevertheless, it was also observed that cultivars derived from certain Asian wild *Vitis* species may also exhibit strong DM resistance [14,15,16]. In 2012 and 2013, two major QTLs, *Rpv10* and *Rpv12*, were identified in the grapevine cultivars ‘Solaris’ and ‘Kozma 20-3’, both descending from accessions of the Asian species *Vitis amurensis* (Rupr.) as resistance donors [15,16]. Originally, Russian and Hungarian breeders were exploiting the strong cold resistance of *V. amurensis* cultivars [17,18]. The additional observation of a resistance to DM in certain accessions of this species made the Asian *Vitis* genus attractive to introgress this trait into the susceptible *V. vinifera* varieties [19]. It is still unknown how *V. amurensis* acquired its resistance against *P. viticola*. Suggestions have proposed a coevolution between the Asian *Vitis* species and the formerly present *Plasmopara* species related to *P. viticola* [20,21]. Despite the different geographical origins, resistance-associated QTLs like *Rpv3* from American, or *Rpv10* and *Rpv12* from Asian wild, grapevines mediate similar defense reactions that are reminiscent of a “hypersensitive response” (HR) [22,23].

Furthermore, genetic similarities in the relevant genomic regions reveal a richness of genes encoding nucleotide-binding site (NBS), leucine-rich repeat (LRR) proteins [10,16,24]. The NB-LRR receptors (NLR) represent a large family of immune receptor proteins [25] that are responsible for pathogen perception and signaling, leading to cell death at the infection site upon host–pathogen interaction [26,27]. The DM resistance found in Asian wild species could also be explained by a wide repertoire of various resistance mechanisms against pathogens. Fan et al. [28] showed a great richness of fungal species colonizing the leaves of *V. amurensis* ‘Shuangyou’, whereof the strains of *Alternaria* could have antagonistic effects on *P. viticola* [28,29].

The three loci *Rpv3*, *Rpv10*, and *Rpv12* have consistently demonstrated strong and reliable resistance to DM across various grapevine varieties and environmental conditions throughout the history of grapevine breeding. Extensive studies and a thorough characterization of these loci have led to the development of reliable markers for marker-assisted selection, rendering these loci as easy to use in a modern breeding program [23,30]. However, new strains of *P. viticola* have recently emerged, which are able to overcome some of the currently used resistances, highlighting the need for new, well-characterized resistances that can be used by grapevine breeders in their breeding programs [30,31].

In this work, the identification of a major QTL responsible for the downy mildew resistance of breeding line We 90-06-12 is presented. The locus is located at the same position as the previously described *Rpv10* but shows differences on the sequence level compared to the *Rpv10* identified in ‘Solaris’ [15]. This may suggest the presence of an allelic variant of *Rpv10*. Such multi-allelism of resistance QTL was observed previously in the case of *Rpv3* [32].

## 2. Results

### 2.1. The Phenotype of Resistance of We 90-06-12

**Figure 1:**
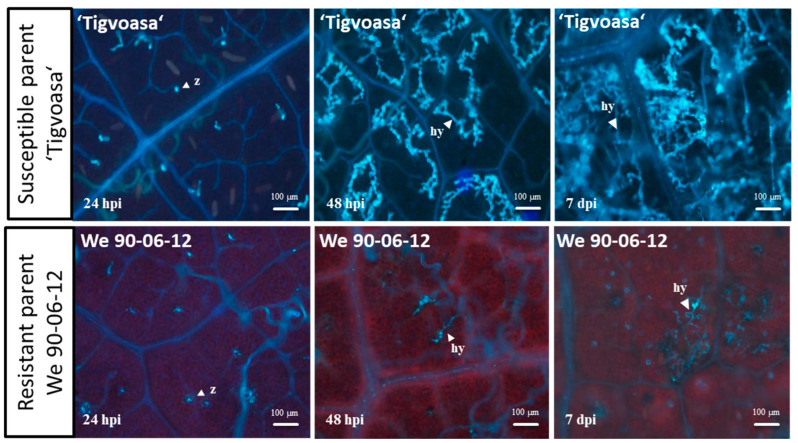
Mycelial development in the leaf discs of the parental lines ‘Tigvoasa’ and We 90-06-12. The infections with zoospores of *P. viticola* after 24 hpi, 48 hpi, and 7 dpi stained with alkaline aniline blue are shown. The white arrows indicate the zoospores (z) and hyphae (hy). The mycelial growth of *P. viticola* was strongly inhibited by We 90-06-12. Scale bar correlates to 100 µm.

To characterize the resistance of the parental lines employed for genetic mapping and QTL analysis, a microscopic investigation on the mycelial development of the infected leaves of ‘Tigvoasa’ (susceptible parent) and We 90-06-12 (resistant parent) was performed. Leaf discs of both the parents were stained with aniline blue after infection with *P. viticola* and observed by fluorescence microscopy at different time points after inoculation (Figure 1). The histological results clearly indicate a resistant phenotype for We 90-06-12.

The hyphal extension was strongly inhibited after primary infection of We 90-06-12. Only a few primary hyphae of the pathogen could extend slightly at 48 hpi. In contrast, a wide network of mycelium was built in the case of leaf discs of ‘Tigvoasa’ 48 hpi. In addition, at 7 dpi, the abaxial growth of sporangiophores became visible (macroscopic).

### 2.2. Segregation of the Phenotypes

**Figure 2:**
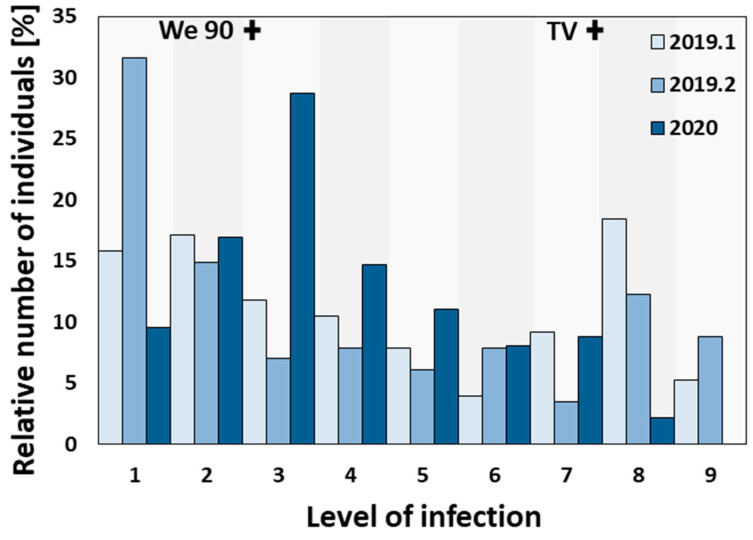
Distribution of the *P. viticola* leaf disc infection tests. The distributions of three leaf disc assays with *P. viticola* with 76 individuals (2019.1), 114 individuals (2019.2), and 136 individuals (2020) are shown. The phenotype was scored inversely to OIV-452 (OIV 2009). The infection levels of ‘Tigvoasa’ (TV) and We 90-06-12 (We 90) in each leaf disc test performed are depicted in the accompanying chart (shown as crosses).

In order to obtain the phenotypic data needed for mapping downy mildew resistance, leaf disc assays were performed. In Figure 2, the results of three independent leaf disc infection tests show the distribution of the resistance levels (1: resistant and 9: susceptible) of the F1 individuals against *P. viticola* (data in Table S4). In addition, the mean scores of the infection level of the parental cultivars ‘Tigvoasa’ (7.4) and We 90-06-12 (2.6) in each independent leaf disc test are indicated below. The leaf disc tests were performed in the middle of July 2019 (2019.1 and 2019.2) and at the end of April 2020. Infection tests 2019.1 and 2019.2 illustrate a bimodal distribution with both a peak in the region of low infection level (1–3) and a peak at high infection level (7–8). In 2020, most individuals (about 55%) showed a low infection level (1–3) and were, in general, resistant to *P. viticola*. To demonstrate consistency and reliability, despite the annual differences of environmental factors such as temperature, humidity, and pathogen virulence, the mean phenotypic data generated in 2019 was correlated with that from 2020. Using a Spearman correlation test (RStudio 3.6.1, [33]), the datasets of 2019 and 2020 showed a positive correlation (*r_SP_* = 0.587) at the significance level of α = 0.01 (Table S4).

### 2.3. Genetic Map

**Figure 3:**
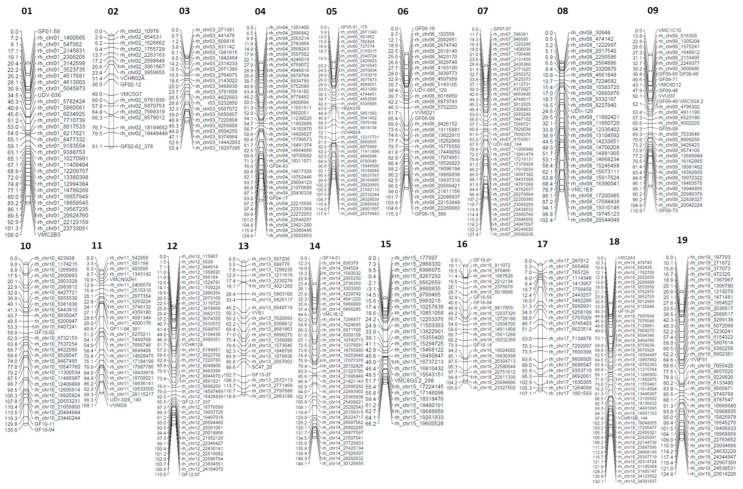
Integrated genetic map of the TV × We 90 linkage groups showing the 19 linkage groups with their respective markers. The markers used for the calculation of the map are a mixture of SSR and rhAmpSeq markers. 01–19 represent the total number of linkage groups.

The marker data of SSR and rhAmpSeq analysis were used to create parental and integrated genetic maps of the cross population “2017-204”. Figure 3 displays the integrated map, which was calculated using 627 SSR and rhAmpSeq markers in total. The map consists of 19 linkage groups and covers a distance of 2107.7 cM. The average marker distance was 3.3 cM.

Further data can be examined in Table 1, which provides an overview of both the integrated map and the parental maps. The calculated parental map of ‘Tigvoasa’ extends over 1869.8 cM, with an average marker distance of 4.6 cM and a total of 421 markers. In the case of We 90-06-12, 539 markers were mapped with a marker distance of 4.2 cM. The whole genome covered a distance of 2203.1 cM. Parental maps are presented in Figures S1 and S2.

### 2.4. QTL Identification

**Figure 4:**
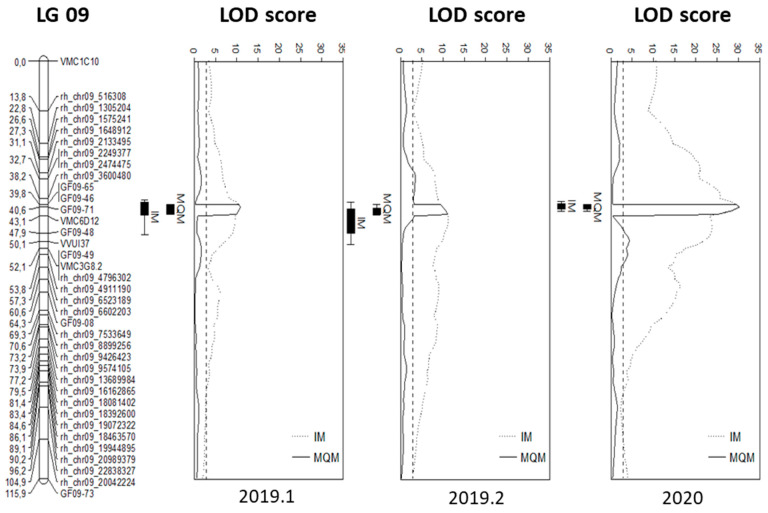
QTL analysis of the *P. viticola* resistance trait. The analysis was performed in IM (dotted) and MQM (consistent) calculations. A resistance correlating QTL was mapped to the same position on LG09 for each of the phenotypic evaluations. The threshold significance for LG09 was calculated at 3.0 for 2019.1 and at 2.9 for 2019.2 and 2020. The genome-wide thresholds were at 4.5 in experiments 2019.1 and 2020 and at 4.7 in 2019.2 (not shown in the diagrams).

Firstly, the QTL identification used Interval Mapping (IM) based on an integrated map of TV × We 90. A significant QTL was detected on LG 9 (Figure 4) and confirmed by analysis on the parental map of We 90-06-12 (Figure S3b). Table 2 shows the LOD scores and corresponding positions on LG 9, which were calculated by IM for the data of 2019.1, 2019.2, and 2020.

For the following MQM, SSR marker GF09-71 was selected as a cofactor. In the case of all three independent tests, the QTL area was restricted to about 3 cM between markers GF09-65 and VMC6D12. In 2020, a maximum LOD score of 30.24 at 40.64 cM was calculated, which explains about 64% of the variance in the phenotype. In 2019, both independent phenotyping tests showed a QTL with a LOD score of around 10, explaining between 35% (2019.2) and 48.7% (2019.1) of the phenotypic variance of the population. In all cases, the calculation with the maternal map of ‘Tigvoasa’ did not result in any significant QTL.

Fine mapping of the QTL was achieved by SSR marker analysis of several recombinants on LG 9 of population TV × We 90 (Table 3). Taking together the data from MQM mapping and the fine mapping approach, the final QTL size was limited to around 80 kb when transferred to the PN40024 genome 12x.V2 reference sequence, and it was flanked by the markers GF09-65 (3.62 Mb) and GF09-47 (3.7 Mb). The QTL was co-localized with the *Rpv10* locus, which was identified in ‘Solaris’ by Schwander et al. [15], and the candidate genes in this area coincided.

Next to an ankyrin-repeat-containing protein (*Vitvi09g00321*), RuBisCo large subunit-binding protein (*Vitvi09g00322*), cytochrome c oxidase assembly protein (*Vitvi09g01591*), CHCH-LETM1-like protein (*Vitvi09g0592*), VHS domain-containing protein (*Vitvi09g00326*), a zinc phosphodiesterase (*Vitvi09g00327*), an AP2/ERF-like protein (*Vitvi09g00323*), and a RPS5-like protein (*Vitvi09g01593*) were declared as candidate genes correlated to an infection by *P. viticola* [15,22,34] (Zyprian et al. in preparation).

### 2.5. Differences between Rpv10 and Rpv10.2

In order to find out if the mapped locus was identical to the one published as *Rpv10* by Schwander et al. [15], the allele lengths of markers in and around the mapped QTL were compared in the resistant breeding lines derived from the *Rpv10.2* carrying *V. amurensis* accession and the several varieties known to carry the previously characterized *Rpv10*. In Table 4, differences in the marker profile of *Rpv10* and the *new QTL*-carrying cultivars are shown for the relevant area, and the close relationship between these loci is illustrated. Therefore, we designated the QTL presented in this work *Rpv10.2* to distinguish it from the *Rpv10* locus.

As the data in Table 4 indicate, the *Rpv10.2* and *Rpv10* carriers differed in allele sizes of the SSR markers GF09-68, GF09-46, and GF09-48. These differences of 2 bp were repeatedly measured. In all other cases, the fragment lengths were uniform. Additionally, the results show that the new German cultivar ‘Sauvitage’ also harbors the *Rpv10.2* locus.

### 2.6. Sequence Comparison of Rpv10 Candidate Genes

Using the current version of the *Vitis vinifera* genome (PN40024 12X.v2) and its corresponding annotations (VCost.v3; [35]), several potential candidate genes were identified in the narrowed QTL region (between 3.62 Mb and 3.7 Mb) on LG 09 (Zyprian et al., in preparation). By isolating, cloning, and sequencing, the variants of two putative candidate genes, *RPS5-like* and *AP2/ERF-like*, from We90-06-12 and from ‘Solaris’, were compared (Figures S3 and S4). Interestingly, no differences were observed in the sequences of both *RPS5-like* candidate genes, including their putative promotor and terminator regions (Figure S3). However, several differences between the *AP2/ERF-like* genomic sequences of We 90-06-12 and ‘Solaris’ were identified. In the promotor region, one single bp deletion and a deleted three bp sequence, as well as four substitutions and one bp insertion, were found in We 90-06-12 compared to ‘Solaris’. Most prominently, an inserted stretch of 18 bp was detected in the ORF region of *AP2/ERF-like* in We 90-06-12, as well as in one substitution (Figure S4). Together with the different allele sizes of some SSR markers, this illustrates that there is a significant amount of diversity between the sequences of *Rpv10* haplophases of We 90-06-12 and ‘Solaris’.

## 3. Materials and Methods

### 3.1. Plant Material

We 90-06-12 (‘Cabernet Sauvignon’ (*V*IVC 1929) × We 73-45-84), an offspring of the Asian wild species *V. amurensis* in a second generation (pBC1)—developed at the LVWO, Weinsberg, Germany—was observed to exhibit a strong resistance (an average score of 2 on an inverted OIV 452 scale, evaluated from 2002–2017) to DM in the field at the LVWO Weinsberg over several years. A cross between the DM-susceptible female *V. vinifera* cultivar ‘Tigvoasa’ (*V*IVC 17114) and the resistant breeding line We 90-06-12 as a pollen donor in 2017 resulted in the F_1_ population “2017-204” consisting of 642 individuals. The cross was performed in the frame of a cooperation between LVWO Weinsberg and JKI (located in southwest Germany). The plants were cultivated in a greenhouse at the JKI Institute for Grapevine Breeding, Geilweilerhof (potted ungrafted plants without fungicide treatments). The growing conditions in the greenhouse were influenced by natural sunlight and the environmental conditions outside. Although it was not possible to precisely control all the parameters, the typical conditions in the greenhouse were as follows: Daytime temperatures ranged from 24 to 30 °C, while night-time temperatures varied between 18 and 22 °C. Relative humidity was between 50 and 70%. The plants were exposed to a natural photoperiod of 12–16 h of light followed by 8-12 h of darkness, with the only light source being natural sunlight.

### 3.2. Phenotyping

Experimental DM inoculations of the leaf discs from the F_1_ plants were performed to display the degree of resistance. In 2019, a first leaf disc test with 76 individuals was conducted, followed by a re-run using 114 individuals. A total of 136 individuals were studied in a leaf disc test in 2020. After initial mapping, 17 individuals were identified as recombinants and underwent additional phenotypical evaluation in 2020 and 2021. Parental lines, as well as the susceptible *V. vinifera* cultivar ‘Müller-Thurgau’ (*V*IVC 8141) and resistant cultivars like ‘Regent’ (*V*IVC 4572, *Rpv3.1*) or ‘Solaris’ (*V*IVC 20340, *Rpv10*), were used as controls. For the tests, the second and third leaf from the shoot tip of each plant were collected, and four discs of a 15 mm diameter were excised with a cork borer. The discs were placed upside down on wet filter paper in square Petri dishes (Nunc^TM^ Square BioAssay Dishes, Thermo Fisher Scientific). As a source of inoculum, infected leaves from different susceptible cultivars were collected in the field every year and stored at −20 °C or immediately used for the experiment. The sporangia were brushed and rinsed off the leaves and dissolved in deionized (d) H_2_O. The sporangial inoculum underwent a vitality test using trypan blue (0.5% trypan blue in a 0.9% NaCl solution) by mixing 36 µL of staining solution with 64 µL of sporangial suspension [36]. Afterward, 40 µL of the suspension (10,000–20,000 sporangia per mL) were applied on each leaf disc. To increase humidity, the square Petri dishes were packed in plastic bags and incubated for 4–6 days at room temperature (22–25 °C). The setup was placed on the laboratory windowsill, which ensured a photoperiod of about 12–14 h. The degree of infection of every leaf disc was scored daily in a scale reverse to the international OIV-452 scale (1: very low, 3: low, 5: medium, 7: high, and 9: very high).

The mean score of the three leaf discs was calculated for each individual genotype for further data analysis. In addition, the hyphal growth was investigated at different time points (24 h post inoculation (hpi), 48 hpi, and 7 days post inoculation (dpi)) in the leaves of the parental genotypes. For this purpose, the experimentally inoculated leaf discs were bleached for up to 3 h with 1 N KOH at 65 °C and stained with aniline blue (0.05%) in K_2_HPO_4_ (0.067 M) for 10 min [37,38]. The treated leaves were washed with dH_2_O, and the stained hyphae were evaluated by fluorescence microscopy (DM4000B, Leica I3, Excitation λ = 450–490 nm, dichroitic mirror 510 nm, emission: LP 515 nm).

### 3.3. DNA Extraction

Tissue fragments of about a 1–2 cm^2^ surface area were collected from the young leaves and stored in pre-cooled 96 deep well plates (Abgene^TM^ 1.2 mL DeepWell Plates, Thermo Fisher Scientific). The samples were lyophilized (Alpha 1–4 LSCbasic, Christ) and crushed in a paint shaker (SK350, Fast & Fluid) with the addition of small metal beads of a 3 mm diameter. DNA was isolated using an extraction kit (NucleoSpin^TM^ Plant II, Macherey-Nagel) and stored at −20 °C in Elution Buffer PE (5 mM Tris/HCL, pH 8.5).

### 3.4. Microsatellite Analysis

Microsatellite analysis was performed using SSR flanking primer pairs in multiplex polymerase chain reactions (MPX-PCR). Thanks to previous work, it was possible to use a comprehensive pool of SSR marker sets like GF [15]; UDV [39]; VCHR [40]; VMC (Vitis Microsatellite Consortium, Agrogene); VRZAG [41]; VVI [42]; VVMD [43,44]; and VVS [45]. Additional primer pairs were designed for the linkage group (LG) 9: Gramene [46], and NCBI Primer-BLAST [47] were used to find SSRs based on the *V. vinifera* PN40024 reference genome 12x.V2 sequence [35]. The primer design was performed using the Eurofins Genomics primer design tool. A full list of the SSR markers used for generating the genetic map can be found in Table S1, and details on the previously unpublished SSR markers used in this study can be found in Table S5. For all the PCR assays, fluorescent dye labels (TAMRA, ROX, HEX, 6-FAM) were linked to the 5′-end of the forward primers (Metabion, Planegg, Germany). MPX-PCRs were realized with up to seven primer pairs in one reaction mix. Reactions were conducted in 384-well PCR plates (ABI type, Biozym) applying Qiagen^®^ Multiplex PCR Kit standards in 5 µL batches containing about 1 ng of sample gDNA and 3 pmol of forward and reverse primer, respectively. The amplification was started with a denaturation phase of 180 s at 95 °C; continued with 30–35 cycles of denaturation (15 s at 95 °C), primer annealing (30 s at 60 °C), and elongation (30 s at 72 °C); and ended with an elongation phase of 560 s at 72 °C. Then, 1 µL of the amplification product was diluted in a 1:2 ratio with dH_2_O and analyzed by capillary electrophoresis (Genetic Analyzer ABI PRISM 3130xl, Applied Biosystems) using a size standard like the GeneScan™ 500 LIZ^®^ (Applied Biosystems). Fragment lengths were recorded with GeneMapper^®^ (Version 5, Applied Biosystems). In addition to SSR markers, rhAmpSeq (RNase H2-dependent amplicon sequencing) was employed [48,49]. For this purpose, freshly extracted and purified DNA was transmitted on ice in 96-well plates to the service provider. The rhAmpSeq markers used for genetic mapping are presented in Table S2. The cultivars in Table S3 were analyzed for their SSR profile at Locus *Rpv10*.

### 3.5. Linkage Mapping

JoinMap^®^ 4 and 5 software (Kyazma B. V, Wageningen, Netherlands) was used to generate parental and integrated maps of the cross population “2017-204” with 244 F_1_ individuals. Linkage groups (LGs) were generated with the cross population (CP) model depending on the recombination frequency of all markers [50]. A groupings tree was calculated, choosing independent LOD scores in a range from 2 to 10 in 1-LOD steps as the parameters. The recombination frequency threshold was set to a maximum of 0.4, and the Kosambi mapping function was applied to convert the recombination frequencies into map distances. Markers with suspect linkages were discarded before map calculation. For map construction, a maximum likelihood algorithm was employed with default parameters, including a jump threshold of 5.0 and a ripple tolerance of 1.0. Additionally, single markers that showed multiple possible linkages, but which were grouped into incorrect LGs (based on the reference genome PN40024 12x.V2), were manually regrouped to the expected LGs if the strongest cross-link information was consistent with the known genetic linkage.

### 3.6. QTL Analysis

MapQTL^®^ 6 software (Kyazma B. V.) was applied to search for the QTL linked to *P. viticola* resistance. The number of individuals utilized in each independent QTL analysis performed depended on the availability of the phenotypic data. Initially, regression-based Interval Mapping (IM) was conducted using the Kosambi mapping function to convert recombination frequencies into map distances. Marker intervals were set at 1 cM for scanning the genome, and a window size of 10 cM was used to exclude neighboring markers during the analysis. After identifying the QTL flanking markers, multiple QTL mapping (MQM) was performed to further refine the QTL regions. Cofactors were selected using a forward-backward stepwise regression method to control the background genetic variation. The LOD score was calculated at 1 cM intervals across the genome, with a significance threshold for LOD values determined by performing a permutation test with 1000 iterations (α = 0.05). A QTL was considered significant if its LOD score exceeded the genome-wide threshold obtained from the permutation test, and confidence intervals for each detected QTL were determined using the 1-LOD drop method.

### 3.7. Sequence Analysis of the Rpv10 Candidate Genes

In order to compare the haplotypes on the sequence level, two genes in the *Rpv10* locus were chosen for sequence analyses. These genes were selected based on previous research on *Rpv10* (‘Solaris’) [15,22,34] (Zyprian et al. in preparation). Subsequently, the genes *RPS5-like* and *AP2/ERF-like* from the two resistant *Rpv10* carriers (‘Solaris’ and We 90-60-12) were cloned into the pJET 1.2 vector, and the plasmid was transformed into *Escherichia coli* (DH5-Alpha). The subsequent sequence analysis was carried out using cycle-sequencing technology (ABI 3730XL DNA Analyser, Applied Biosystems) by Eurofins Genomics. The primers used for sequencing were designed at a distance of approx. 500 bp from each other based on the sequence of the *RPS5-like* and *AP2/ERF-like* genes from ‘Solaris’ with the sequencing primer design tool by Eurofins Genomics. The insert-flanking primers of the pJET 1.2 vector were used as the start and end points of the sequences (pJET 1.2 Standard Primer GATC, Eurofins Genomics). The sequenced fragments were combined, aligned, and analyzed using the CLC Main Workbench version 21.0.1 software (QIAGEN Digital Insights).

## 4. Discussion

*P. viticola* was introduced to Europe from North America in the late 19th century. It is a grapevine pathogen that causes severe damage to the plants and leads to prominent yield loss if no counteractions are taken [2]. Resistance loci against *P. viticola*, identified in cultivars descending from wild grapevine species, have shown their potential in grapevine breeding. Some North American non-vinifera species, like *Vitis riparia*, *Vitis cinerea*, and *Vitis labrusca*, as well as the Northeast Asian *Vitis amurensis*, exhibit moderate-to-high resistance. To date, 37 *Rpv* loci have been successfully identified. However, only few of them have been well characterized and extensively studied in terms of their resistance mechanisms. The most commonly utilized resistance loci are *Rpv3* and its haplotypes (*Vitis rupestris*) [10,39]. Two of the most important loci for grapevine breeding, namely *Rpv10* and *Rpv12*, originate from the Asian wild species *V. amurensis* and provide strong resistance to DM [15,16]. Recently, researchers discovered further loci named *Rpv22-24* [51] and *Rpv25-26* [52] in the Asian varieties ‘Shuanghong’ and ‘Shuangyou’, which are offsprings of the wild grape *V. amurensis*.

Here, we provide information about a new allelic variant of the *Rpv10* locus. It was identified in a cross population with *V. amurensis* in the pedigree. The QTL analysis identified a highly significant QTL on LG 9 (Figure 4) based on a high-density map generated by the successful combination of SSR and rhAmpSeq markers (Figure 3). While rhAmpSeq markers were used to increase the map density, SSR markers showed their relevance, especially in fine mapping. The identified QTL was determined to be a haplotype variation of the previously identified *Rpv10* locus by a small but stable difference in the fragment lengths of the well-known *Rpv10* locus-linked markers GF09-46 and GF09-48 (Table 3). Both markers can be used to distinguish the loci in marker-assisted selection and are suitable options for tracing the *Rpv10.2* locus in grapevine breeding. A sequence analysis of the two genes present in the *Rpv10.1* and *Rpv10.2* loci further strengthened the idea that We 90-06-12 carries a haplotypic variant of *Rpv10*. The Asian wild species *V. amurensis* seems to harbor a broad diversity of resistance genes against DM as, after *Rpv12* and *Rpv10*, there has now also been a haplotypic variant of *Rpv10* identified. This highlights the possibility that there is further untapped potential for highly promising resistances against different grapevine pathogens still present in the *V. amurensis* germplasm. The occurrence of different haplotypic variants was already observed for the *Rpv3* locus found in the cultivars descending from North American wild species (e.g., *Rpv3.1* [9], *Rpv3.2* [53], *Rpv3.3* [54]). It is known that North American wild grapevines like *V. rupestris* or *V. riparia* underwent a coevolution with *P. viticola* over a very long time and developed resistance against the pathogen [9,55]. Interestingly, several individual accessions of *V. amurensis* also appear to possess resistance genes against *P. viticola*, even though it is believed that the pathogen did not exist in Asia before its introduction in 1899 [56,57]. The likelihood of a pathogen–host relationship evolving an immune response through coevolution within less than a century is highly uncertain. Consequently, some researchers propose a link between the resistance loci in Asian wild grapes and effector-triggered immunity (ETI) involving nucleotide-binding leucine-rich repeat receptor (NLR) genes. These resistance genes (R genes) have evolved through a dynamic interaction, and these are characterized by a zigzag pattern, where a pathogen secretes an effector or an avirulence protein (avr protein) during host infection events [58]. According to new insights into the ETI-dependent immune response, an active ‘plant resistosome’ is formed by a permanent conformational change in NLRs [59,60], resulting in a HR and programmed cell death (PCD). An NLR gene of the coiled-coil (CC) type called *RPS5-like* was identified as a candidate gene in the *Rpv10.1* and *Rpv10.2* loci [22,49](Zyprian et al. in preparation). Furthermore, an HR-mediated PCD was observed in the leaf infection tests of ‘Solaris’ [61]. Together with our findings, this indicates a successful post-penetration resistance to *P. viticola* due to the *Rpv10* haplotypes by suppressing the hyphal growth of the oomycete inside the leaf (Figure 1). To date, no correlation between *RPS5-like* gene activity and DM resistance has been observed; instead, a constant expression of *RPS5-like* can be inferred [22]. Recent data revealing the presence of approximately 150 fungal endophytes on the leaves of *V. amurensis* ‘Shuangyou’ suggest the existence of resistance in Asian wild grapes, which is not based on ETI but rather depends on an increased quantity of immune responses of the PAMP-triggered immunity (PTI) type [28]. Secondary plant metabolites such as stilbenes and anthocyanins have become important in the grapevine resistance against pathogens. In addition to WRKY and MYB, transcription factors (Tfs) from the AP2/ERF family have been identified as part of the signaling network for immunity in response to the *P. viticola* infection in the ‘Solaris’ grapevine [22]. Further investigation is required to determine whether the candidate genes *RPS5-like* or *AP2/ERF-like*, present in the *Rpv10.1* [15,22,34] (Zyprian et al. in preparation) and *Rpv10.2* loci, are involved in the DM resistance mediated by Rpv10 variants.

Multiple studies have shown that *P. viticola* is able to quickly adapt to resistances and that new isolates have emerged, demonstrating significantly increased sporulation on different varieties carrying resistance genes, such as *Rpv3.1* or *Rpv12* [30,31,62,63,64]. *Rpv3, Rpv10*, and *Rpv12* are commonly used in grapevine breeding due to their proven effectiveness and compatibility. However, due to the aforementioned rapid adaptation, discovering and utilizing new resistance loci and genes with different mechanisms is essential for enhancing and sustaining long-term resistance to DM in grapevines. Diversifying resistance mechanisms will also help to reduce the risk of widespread crop loss if a pathogen isolate overcomes the resistance conferred by the commonly used loci. However, it remains uncertain whether *Rpv10.1* and *Rpv10.2* are mechanistically different from each other. Whether a combination of both loci in breeding would be feasible, therefore, requires further research.

Disease-resistant grapevine cultivars have the potential to minimize the plant protection efforts needed in viticulture, thus making viticulture more sustainable and aligned with political and societal goals. With ongoing climate change, there is an increase in extreme weather events, which often creates favorable conditions for grapevine pathogens in Europe, leading to yield loss due to high pathogen damage. The resistance properties of disease-resistant varieties may enhance yield security for the winemaker, especially in difficult weather conditions. Additionally, disease-resistant varieties and a reduction in plant protection can enhance the richness of arthropods in vineyards, which, in turn, may be beneficial in natural pest control [65]. With the characterization of *Rpv10.2*, there is now a novel option in the toolbox of grapevine breeders allowing new approaches to the pyramid resistances against DM in their breeding efforts, and it opens up new possibilities for future resistant cultivars.

## 5. Conclusions

A haplotype variant of the *Rpv10* locus was identified in a *Vitis* cross of ‘Tigvoasa’ × We 90-06-12. This locus, now designated as *Rpv10.2*, was introgressed from the Asian wild grape *V. amurensis* and is co-located with *Rpv10*, which was identified in ‘Solaris’ several years ago. *Rpv10.2* can be applied in future resistance breeding efforts and can be stably distinguished from *Rpv10* by SSR marker analysis, enabling approaches to combine both in grapevine breeding. Furthermore, the identification of *Rpv10.2* promotes new possibilities for investigating the mechanism of *Rpv10*-mediated resistance.

## Figures and Tables

**Table 1:** Linkage group (LG) marker data for the parental maps ‘Tigvoasa‘ (TV) and We 90-06-12 (We 90), as well as the integrated map (I). The average distance between the markers and the total length of the genome are noted in centiMorgan (cM).

LG	No. of Markers			Average Distance [cM]			Total Length [cM]		
	I	TV	We90	I	TV	We90	I	TV	We90
1	33	19	28	3.1	4.4	4.2	106	83.3	118.1
2	18	13	17	4.8	4.3	5	91.1	55.8	84.8
3	22	14	17	2.5	3.2	3.4	55.4	44.7	57
4	40	33	23	3	3.8	5	124.4	123.9	113.8
5	40	21	40	2.7	4.5	2.7	117.6	93.5	107.5
6	34	19	29	3.4	5	3.8	115.3	94.4	111.1
7	42	25	35	2.9	4.8	3.4	127.4	121.1	119.9
8	32	25	28	3.2	3.4	4.2	102.4	86	116.7
9	36	26	31	3.1	4.7	4.3	115.9	122.4	132.4
10	31	27	24	4.2	5.4	5.5	135.6	144.5	132.6
11	28	16	23	3.7	7.2	4.4	108.1	114.5	101.2
12	43	36	32	2.4	4.1	2.8	110	148.9	88.2
13	25	6	29	4.2	8.6	5.1	118.6	51.3	147.7
14	41	33	38	3.2	4.5	4.9	144.1	147.6	185.2
15	28	13	31	2	2.9	3.3	66.2	37.7	101.4
16	25	19	24	3.9	4.7	4.5	105.6	88.9	106.8
17	23	16	22	4.5	5	6.3	107.1	80.3	139
18	47	32	42	2.6	4	2.8	132.1	129.2	119
19	39	28	26	3	3.6	4.6	124.8	101.8	120.7
Σ/$\bar{x}$.	627	421	539	3.3	4.6	4.2	2107.7	1869.8	2203.1
σ				0.76	1.34	0.98			

**Table 2:** The QTL calculated for the integrated map (I) and the parental maps (TV, We 90) using IM. The LOD scores, positions, and phenotypic variance (VE) of the QTL, as well as the linkage group specific significance levels (α = 0.05), are listed.

Map	Test Inoculation	LG	Cofactor	Position [cM]	LOD Score	α = 0.05	VE [%]
I	2019.1	9	GF09-71	40.64	10.86	3	48.7
	2019.2	9	GF09-71	40.64	9.9	2.9	35
	2020	9	GF09-71	40.64	30.24	2.9	64
TV	2019.1	-	-	-	-	-	-
	2019.2	-	-	-	-	-	-
	2020	-	-	-	-	-	-
We 90	2019.1	9	-	51.8	10.95	1.7	49
	2019.2	9	-	55.09	8.05	1.6	29.5
	2020	9	GF09-71	50.08	30.3	1.7	64.2

**Table 3:** Fine mapping and further restriction of the QTL by marker analysis of specific recombinants. The resistance allele could be traced back to the original donor *V. amurensis* (VA) and symbolized by a ‘+’ (green color: presence of *Rpv10.2* allele, peach color: absence) in case of ‘Tigvoasa’ (TV), We 90-06-12 (We90), and the recombinants. In addition, the corresponding downy mildew (DM) infection level based on the inversed OIV 452 scale is listed below. n/a: data not available.

SSR Marker	VMC1C10	GF09-11	GF09-43	GF09-62	GF09-64	GF09-65	GF09-68	GF09-46	GF09-70	GF09-71	GF09-47	VMC6D12	GF09-48	GF09-16	DM
**Physical position Chr.09**	0.56 Mb	2.95 Mb	3.15 Mb	3.5 Mb	3.6 Mb	3.62 Mb	3.67 Mb	3.67 Mb	3.68 Mb	3.69 Mb	3.7 Mb	3.8 Mb	3.85 Mb	5.92 Mb	
**VA *(Rpv10.2) allele size***	150	287	427	383	281	312	153	413	334	366	299	146	357	243	
**We90**	**+**	**+**	**+**	**+**	**+**	**+**	**+**	**+**	**+**	**+**	**+**	**+**	**+**	**+**	
**TV**	**−**	**−**	**−**	**−**	**−**	**−**	**−**	**−**	**−**	**−**	**−**	**−**	**−**	**−**	
**2017-204-12**	**−**	**+**	**+**	**+**	**+**	**+**	**+**	**+**	**+**	**+**	**+**	**+**	**+**	**+**	2
**2017-204-19**	**−**	**+**	**+**	**+**	**+**	**+**	**+**	**+**	**+**	**+**	**+**	**+**	**+**	**+**	3
**2017-204-28**	**+**	**+**	**+**	**+**	**+**	**+**	**+**	**+**	**+**	**+**	**+**	**+**	**+**	**−**	3
**2017-204-67**	**+**	**+**	**+**	**+**	**+**	**+**	**+**	**+**	**+**	**+**	**+**	**−**	**−**	**−**	3
**2017-204-72**	**−**	**+**	**+**	**+**	**+**	**+**	**+**	**+**	**+**	**+**	**+**	**+**	**+**	**+**	3
**2017-204-78**	**−**	**−**	**−**	**+**	**+**	**+**	**+**	**+**	**+**	**+**	**+**	**+**	**+**	**+**	3
**2017-204-94**	**+**	**+**	**+**	**+**	**+**	**+**	**+**	**+**	**+**	**+**	**+**	**+**	**+**	**−**	2
**2017-204-168**	**−**	**+**	**+**	**+**	**+**	**+**	**+**	**+**	**+**	**+**	**+**	**+**	**+**	**+**	2
**2017-204-195**	**+**	**+**	**−**	**+**	**+**	**+**	**+**	**+**	**+**	**+**	**+**	**+**	**+**	**−**	3
**2017-204-240**	**−**	**+**	**+**	**+**	**+**	**+**	**+**	**+**	**+**	**+**	**+**	**+**	**+**	**+**	3
**2017-204-413**	**+**	**−**	**n/a**	**+**	**+**	**+**	**+**	**+**	**+**	**n/a**	**+**	**+**	**+**	**−**	1
**2017-204-423**	**−**	**+**	**+**	**+**	**+**	**+**	**+**	**+**	**+**	**+**	**+**	**n/a**	**−**	**+**	1
**2017-204-630**	**−**	**+**	**+**	**+**	**+**	**+**	**+**	**+**	**+**	**+**	**+**	**+**	**−**	**+**	2
**2017-204-24**	**+**	**+**	**−**	**−**	**−**	**−**	**−**	**−**	**−**	**−**	**−**	**−**	**−**	**−**	6
**2017-204-178**	**−**	**−**	**−**	**−**	**−**	**−**	**−**	**−**	**−**	**−**	**+**	**+**	**+**	**+**	7
**2017-204-209**	**+**	**−**	**−**	**−**	**−**	**−**	**−**	**−**	**−**	**−**	**−**	**−**	**−**	**−**	7
**2017-204-243**	**+**	**+**	**+**	**−**	**−**	**−**	**−**	**−**	**−**	**−**	**−**	**−**	**−**	**−**	6

**Table 4:** Differences in the specific SSR marker allele length between *Rpv10.2*- and *Rpv10*-carrying cultivars. The markers GF09-68, GF09-46, and GF09-48 showed a significant and repeatedly observed difference between the loci *Rpv10* and *Rpv10.2*, while the rest shared identical allele lengths. ‘Cabernet franc’ and ‘Muscat á Petits Grains’, two susceptible cultivars commonly used as controls for genetic fingerprinting in *Vitis*, are listed as the control. n/a: not available. Green color: presence of *Rpv10.2* allele; peach color: absence.

SSR Marker	GF09-62	GF09-64	GF09-65	GF09-68	GF09-46	GF09-70	GF09-71	GF09-47	GF09-48	*Rpv* Allele
Physical Position Chr.09	3.5 Mb	3.6 Mb	3.62 Mb	3.67 Mb	3.67 Mb	3.68 Mb	3.69 Mb	3.7 Mb	3.85 Mb	
***V. amurensis Rpv10.2 allele size***	383	281	312	153	413	334	366	299	357	*Rpv10.2*
**We 73-45-84**	**+**	**+**	**+**	**+**	**+**	**+**	**+**	**+**	**+**	
**We 90-06-12**	**+**	**+**	**+**	**+**	**+**	**+**	**+**	**+**	**+**	
**We 75-103-07**	**+**	**+**	**+**	**+**	**+**	**+**	**n/a**	**+**	**+**	
**We 73-40-27**	**+**	**+**	**+**	**+**	**+**	**+**	**+**	**+**	**n/a**	
**We 75-115-07**	**+**	**+**	**+**	**+**	**+**	**+**	**+**	**+**	**+**	
**We 75-90-03**	**+**	**+**	**+**	**+**	**+**	**+**	**+**	**+**	**+**	
**We 75-14-23**	**+**	**+**	**+**	**+**	**+**	**+**	**+**	**+**	**n/a**	
**We 75-36-26**	**+**	**+**	**+**	**+**	**+**	**+**	**+**	**+**	**+**	
**We 75-34-13**	**+**	**+**	**+**	**+**	**+**	**+**	**+**	**+**	**+**	
**We 75-108-10**	**+**	**+**	**+**	**+**	**+**	**+**	**+**	**+**	**+**	
**‘Sauvitage’**	**+**	**+**	**+**	**+**	**+**	**+**	**+**	**+**	**+**	
**‘Solaris’**	**+**	**+**	**+**	155	415	**+**	**+**	**+**	359	*Rpv10*
**‘Baron’**	**+**	**+**	**+**	155	415	**+**	**+**	**+**	359	
**‘Monarch’**	**+**	**+**	**+**	155	415	**+**	**+**	**+**	359	
**‘Muscaris’**	**+**	**+**	**+**	155	415	**+**	**+**	**+**	359	
**‘Rondo’**	**+**	**+**	**+**	155	415	**+**	**+**	**+**	359	
**‘Cabernet Cantor’**	**+**	**+**	**+**	155	415	**+**	**+**	**+**	359	
**‘Cabernet Carbon’**	**+**	**+**	**+**	155	415	**+**	**+**	**+**	359	
**‘Cabernet Carol’**	**+**	**+**	**+**	155	415	**+**	**+**	**+**	**n/a**	
**‘Cabernet Cortis’**	**+**	**+**	**+**	155	415	**+**	**+**	**+**	359	
**‘Cabernet franc’**	**−**	**−**	**−**	**−**	**−**	**−**	**−**	**−**	**−**	Control
**‘Muscat a Petits Grains’**	**−**	**−**	**−**	**−**	**−**	**−**	**−**	**−**	**−**	

## Data Availability

The datasets generated and/or analyzed in this study are available from the corresponding author on reasonable request.

## Supplementary Materials

The following supporting information can be downloaded at: https://www.mdpi.com/article/10.3390/plants13182624/s1, Table S1: List of the SSR markers used for genetic mapping. The marker name, SSR pattern, linkage group (LG), and allele length of ‘Tigvoasa’ and We 90-06-12 are given. Table S2: List of the rhAmpSeq markers used for genetic mapping. The called alleles were transformed manually into a JoinMap segregation pattern. The marker name, segregation pattern, linkage group (LG), as well as the position of the reference genome PN40024 12x.V2 in [bp] are given. Table S3: The following cultivars were tested for their allelic profile of several *Rpv10*-linked SSR markers. The prime name/cultivar name and the assigned variety number of the *Vitis* international Variety Catalogue (*V*IVC) are given. Table S4: Phenotypical data of the downy mildew infection evaluations used for Spearman correlation (*cor.test(x,y,method = "spearman", use="pairwise.complete.obs")*) in RStudio 3.6.1 [50]. Given are the genotypes, the level of infection according to an inverted OIV 452 scale, the mean value, as well as the standard deviation. Table S5: Details of the unpublished SSR markers used in the genetic analysis. This table includes the names of the SSR markers, their corresponding chromosome locations (Chr.no and Phys. Pos.), and the sequences of the forward and reverse primers for each marker. Figure S1: A maternal genetic map (Tigvoasa) of the population TV × We 90. Figure S2: Paternal genetic map (We 90-06-12) of the population TV × We 90. Figure S3: Illustration of the sequence comparison of the candidate gene variant *RPS5-like* from the varieties ‘Solaris’ (top) and We 90-06-12 (bottom), respectively. The ORF region (782 bp to 3439 bp) is indicated as a yellow bar. Figure S4: Illustration of the sequence comparison of the candidate gene variant AP2-like from the varieties ‘Solaris’ (top) and We 90-06-12 (bottom), respectively. The ORF region (924 bp to 1631 bp) is indicated as a yellow bar.
