# Supplementary figures and images for: Rpv10.2: A Haplotype Variant of Locus Rpv10 Enables New Combinations for Pyramiding Downy Mildew Resistance Traits in Grapevine
Source: Plants (Basel). 2024 Sep 20;13(18):2624. doi: 10.3390/plants13182624 (PMC11434656; doi:10.3390/plants13182624)

## Slide 1
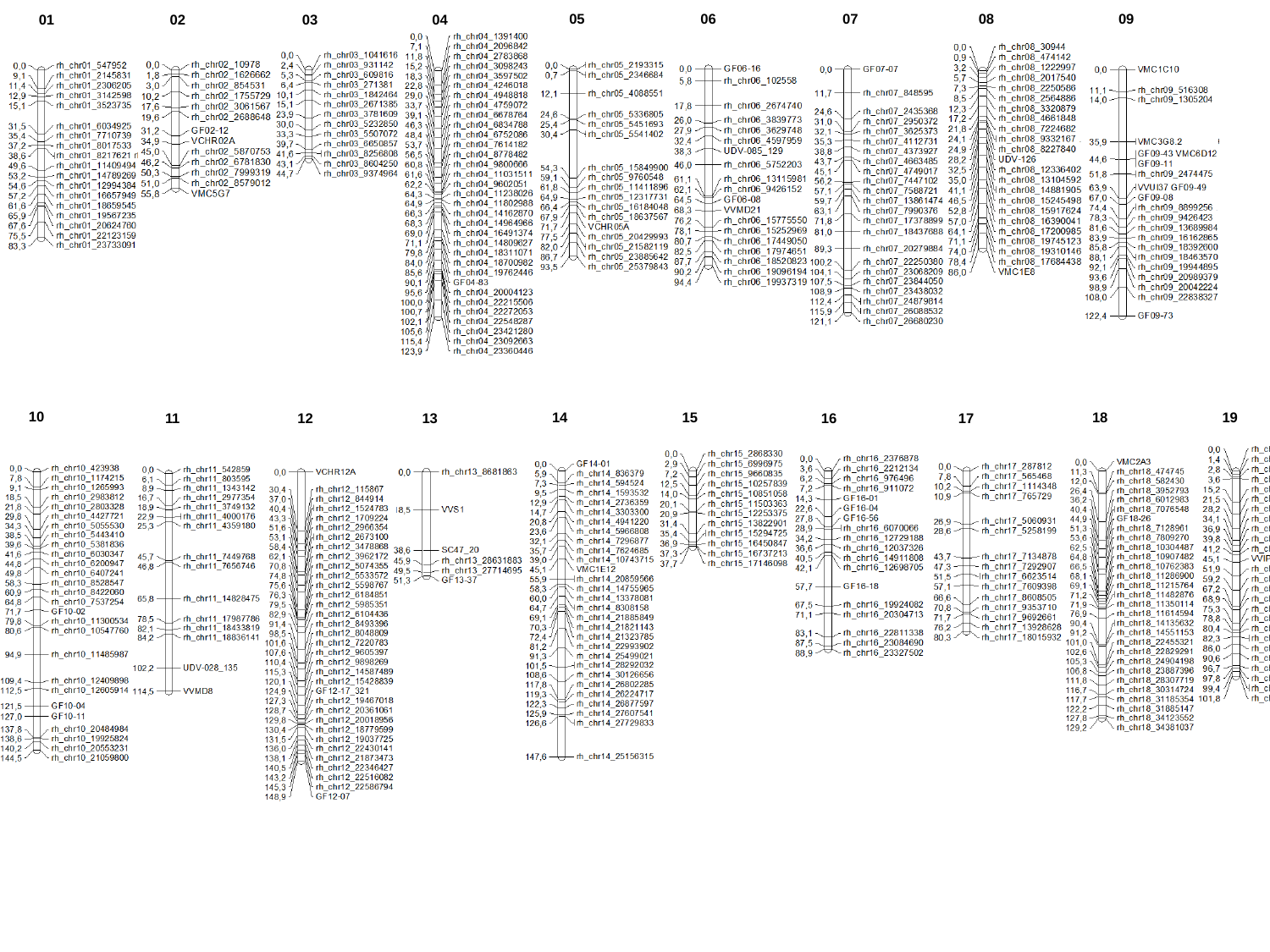

05
09
06
07
08
01
03
02
04
10
19
15
14
18
12
13
11
16
17

Supplement: Supplementary file 1 [file plants-13-02624-s001.zip › Figure S1.pptx]

## Slide 1
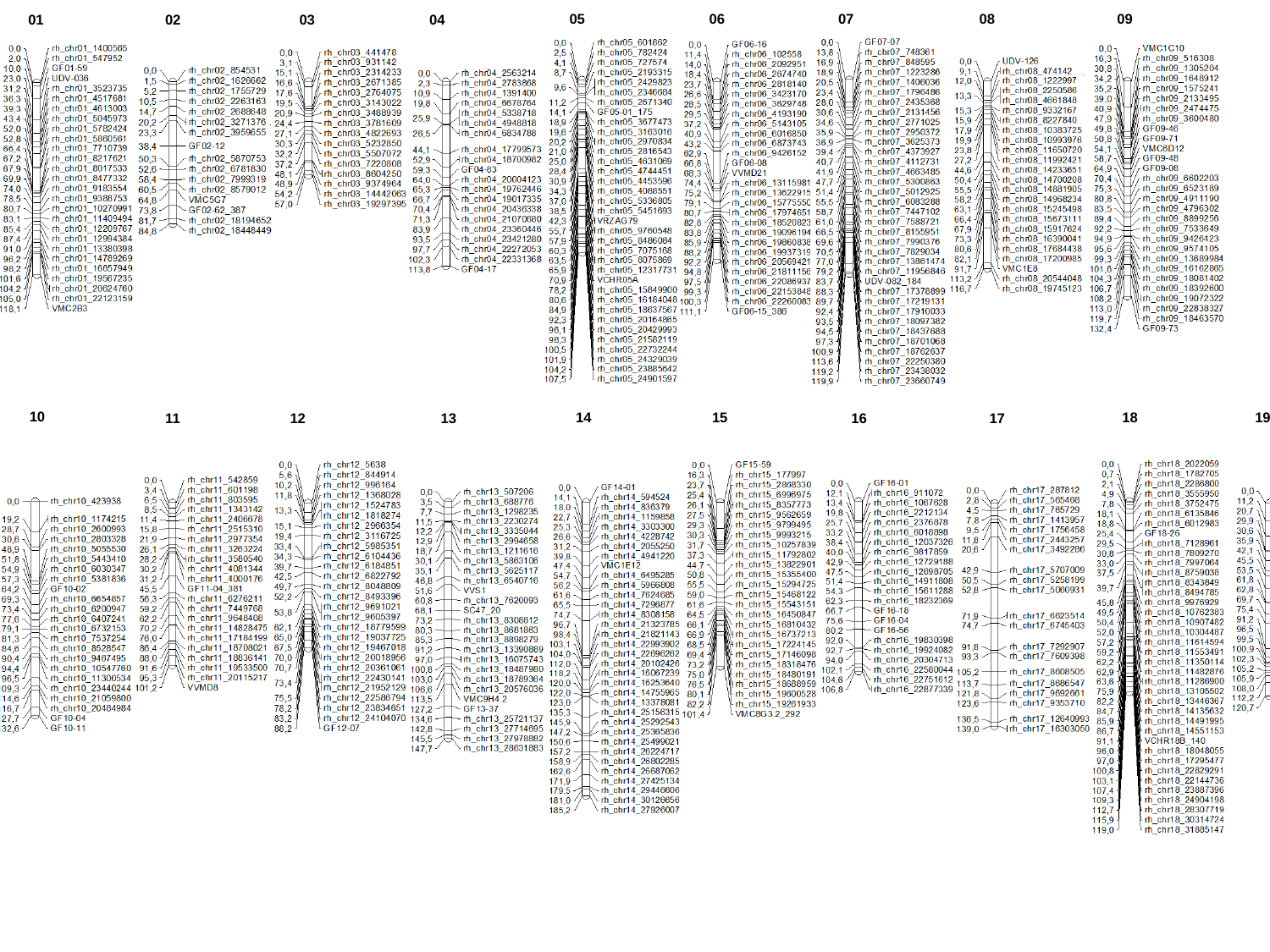

05
09
06
07
08
01
03
02
04
10
19
15
14
18
12
13
11
16
17

Supplement: Supplementary file 1 [file plants-13-02624-s001.zip › Figure S2.pptx]

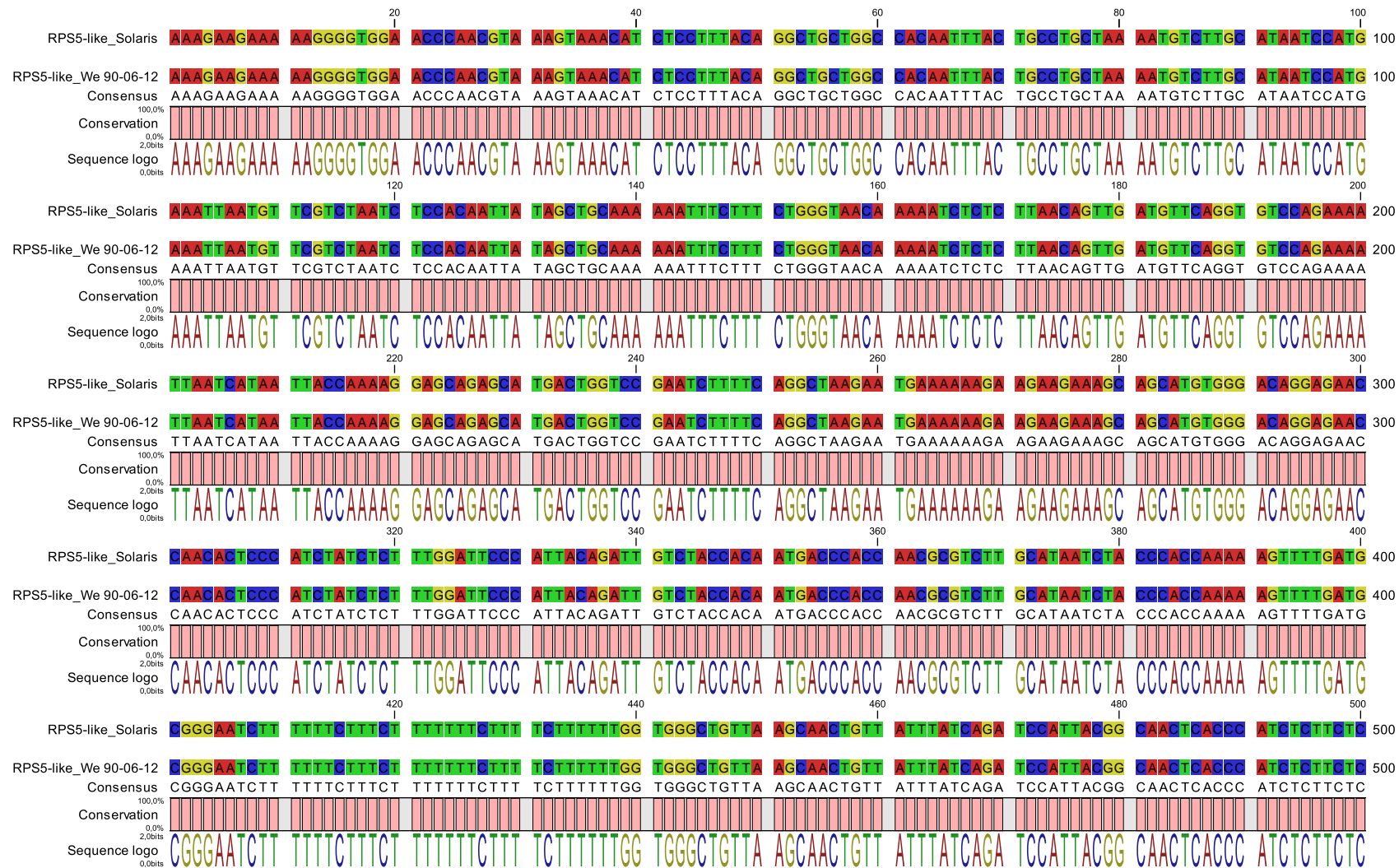

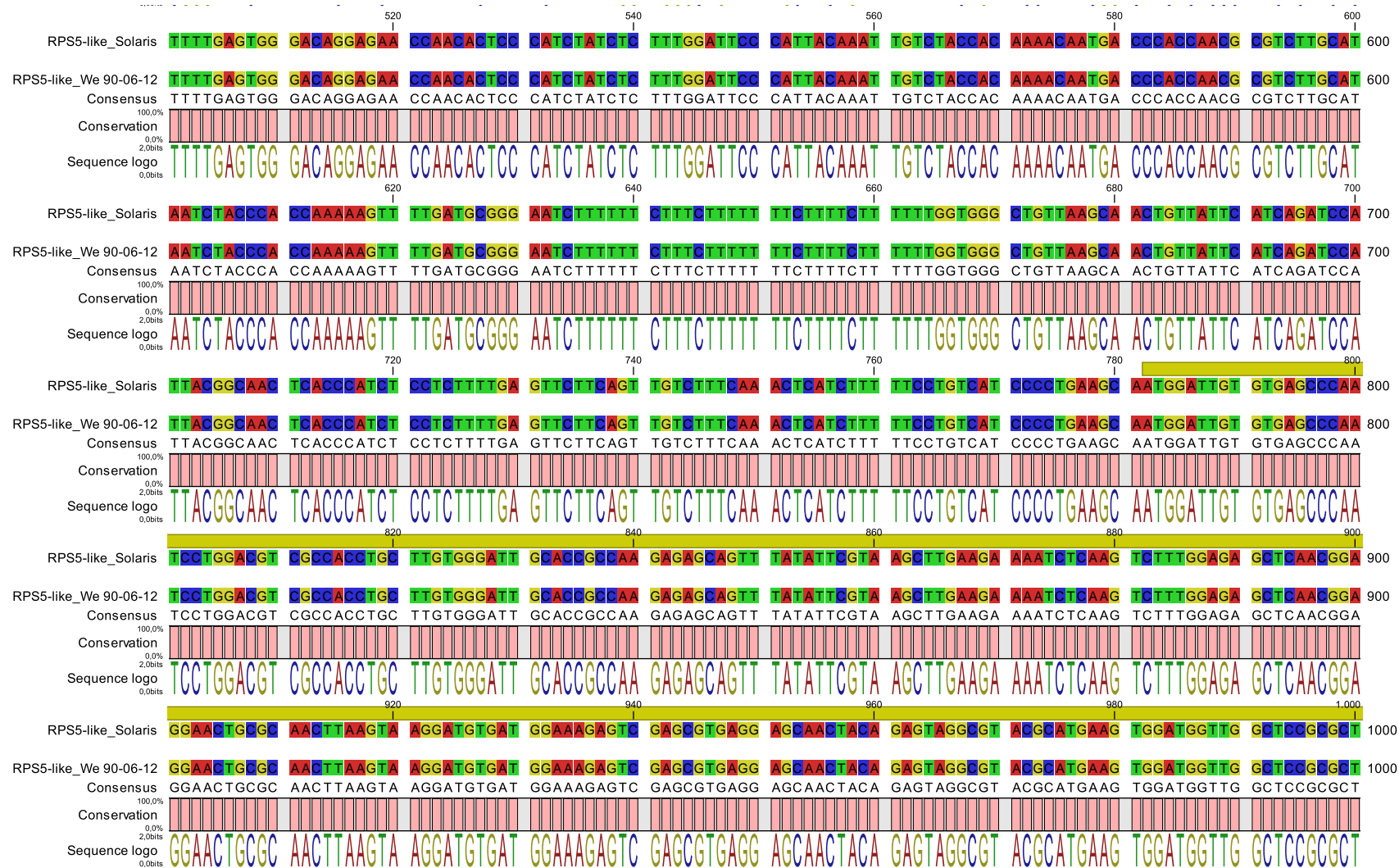

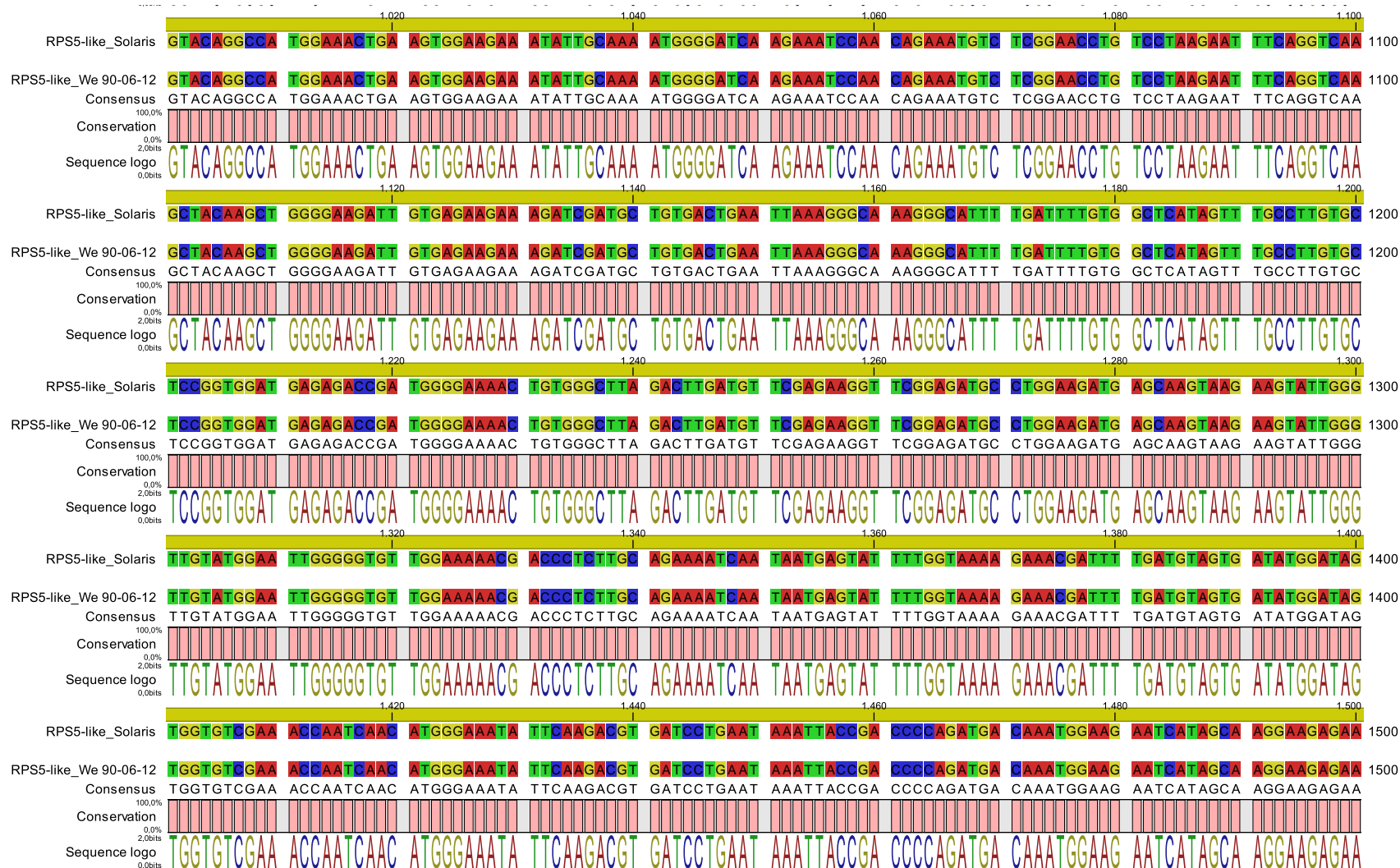

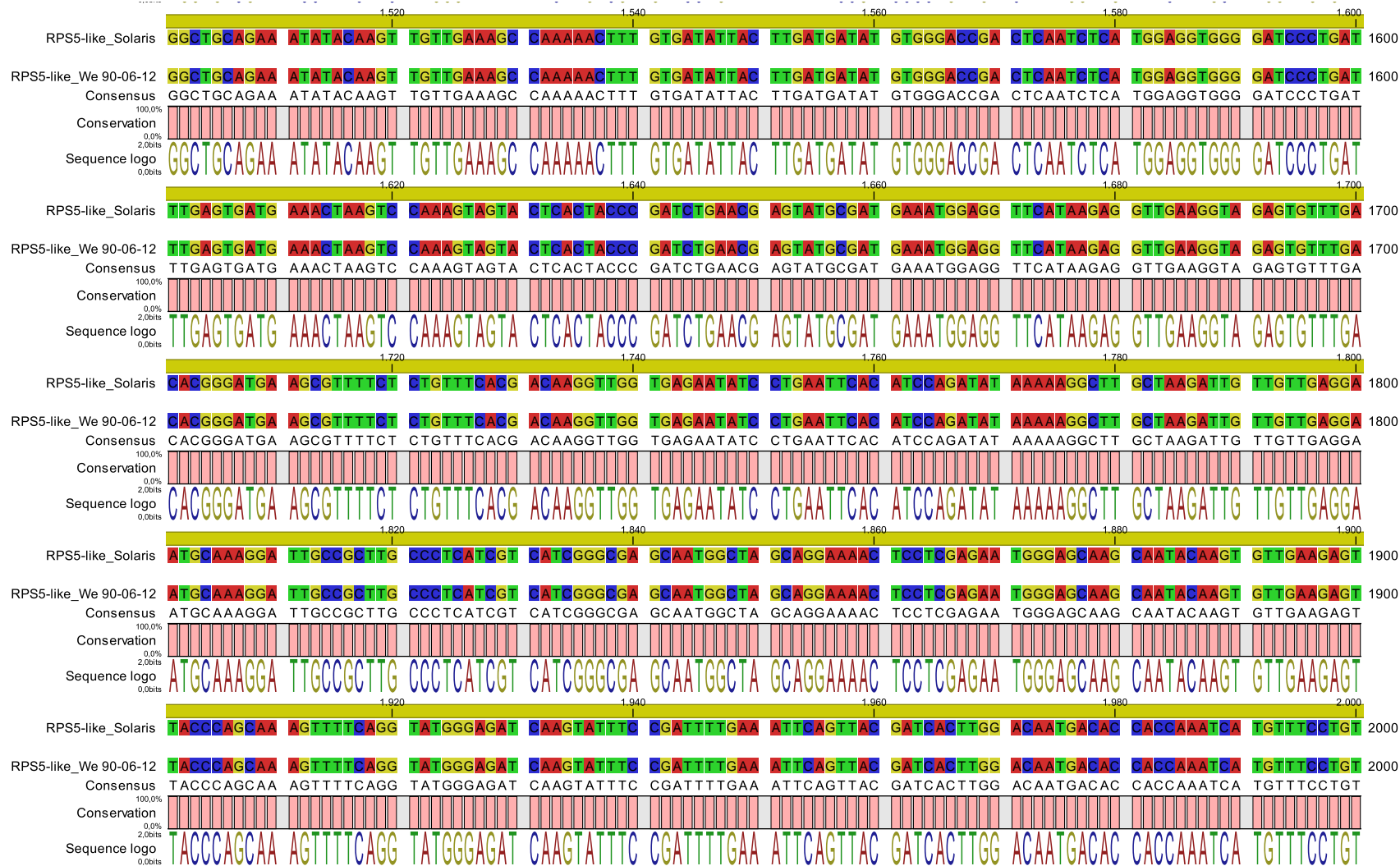

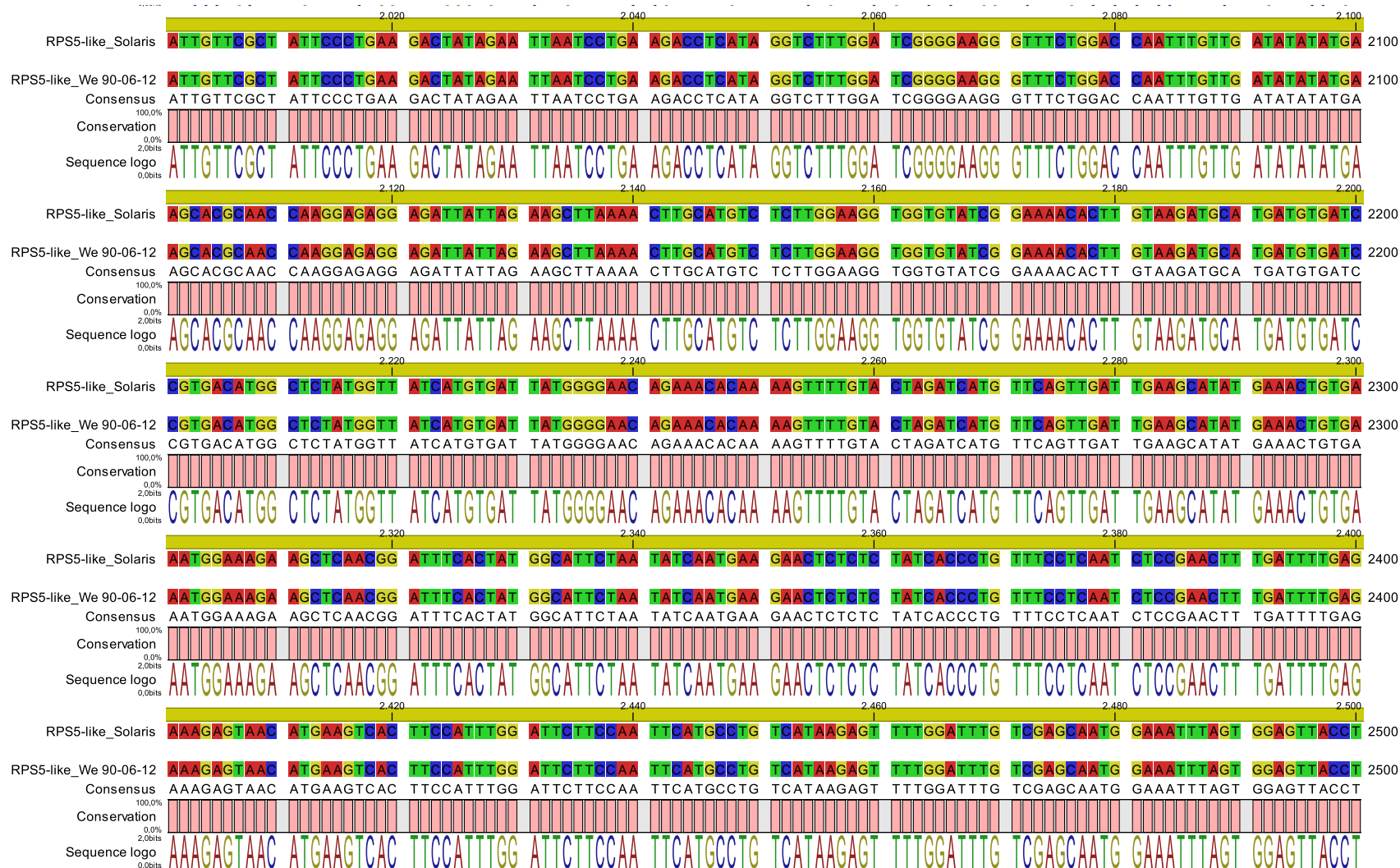

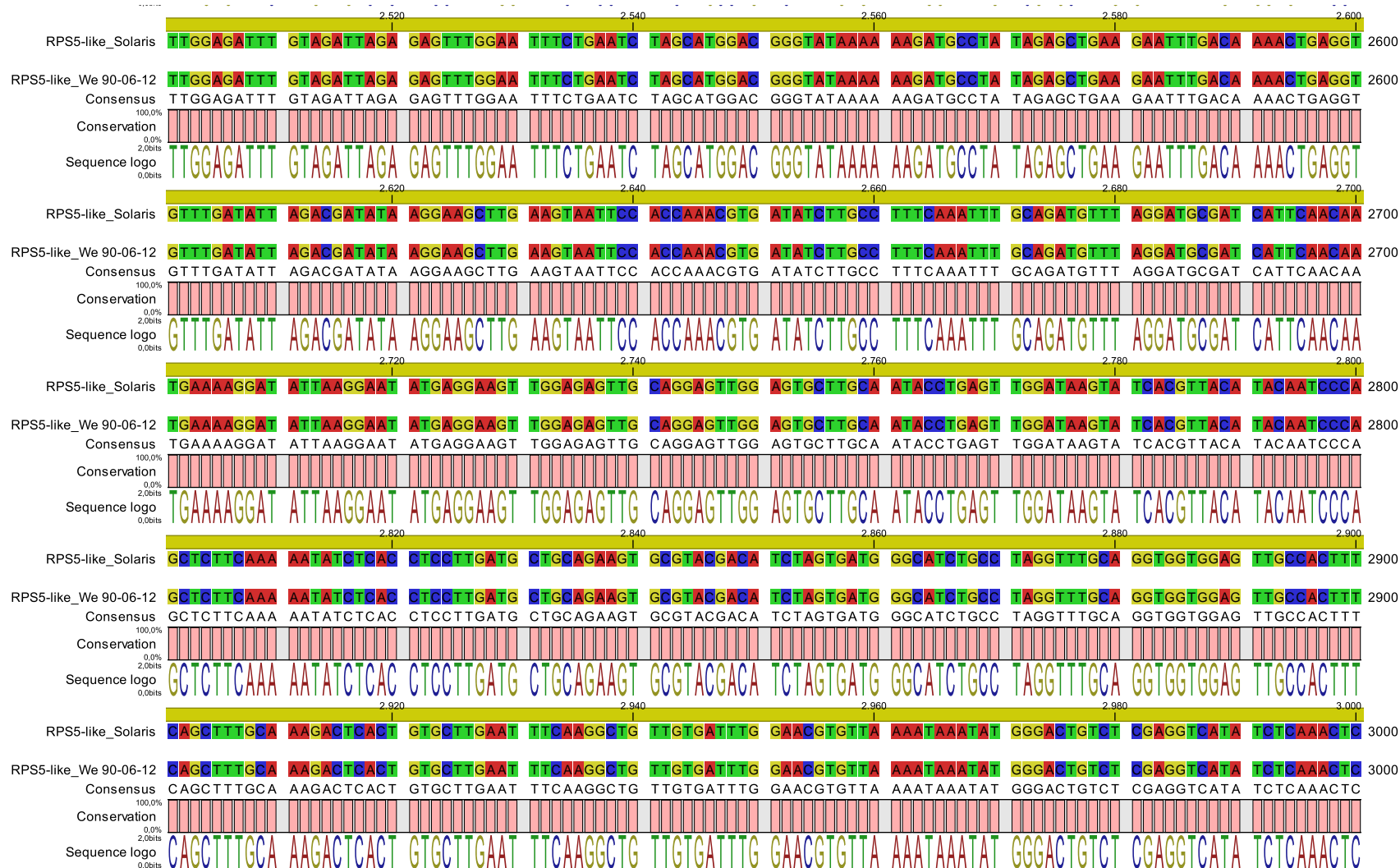

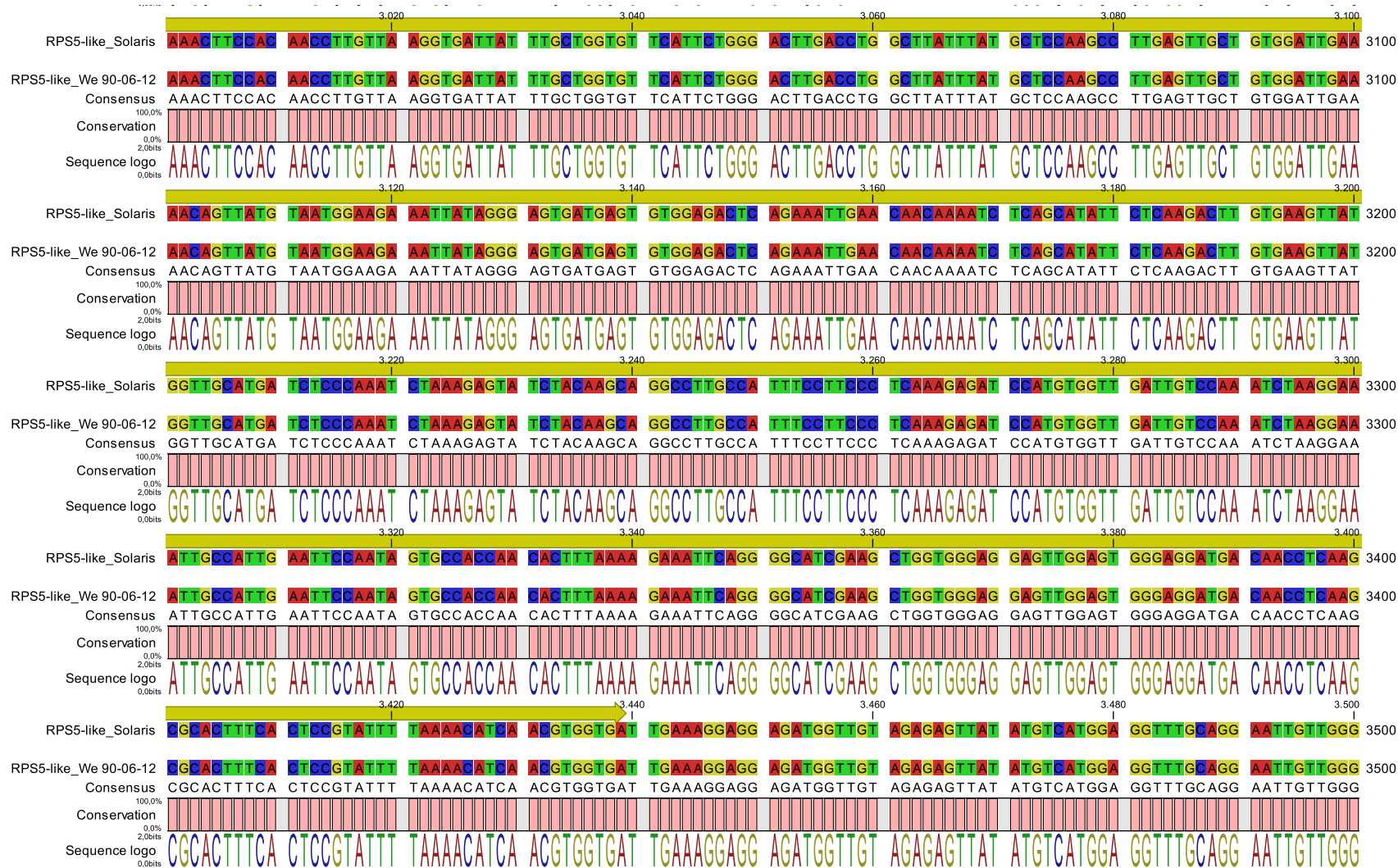

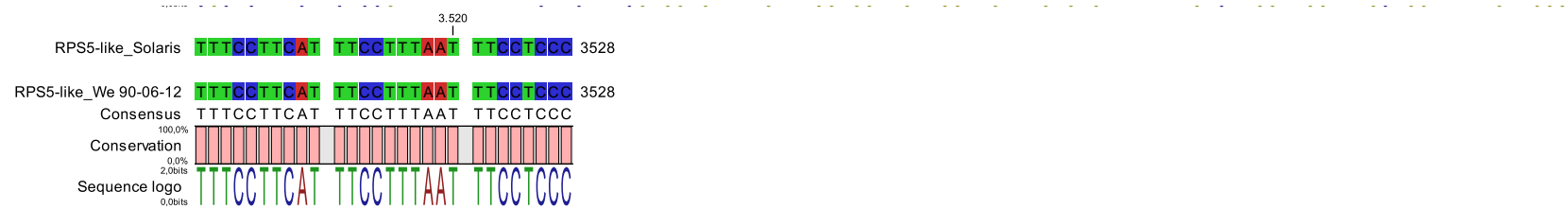

Supplement: Supplementary file 1 [file plants-13-02624-s001.zip › Figure S3_Final_Sol-WE90_RPS5-1.pdf]

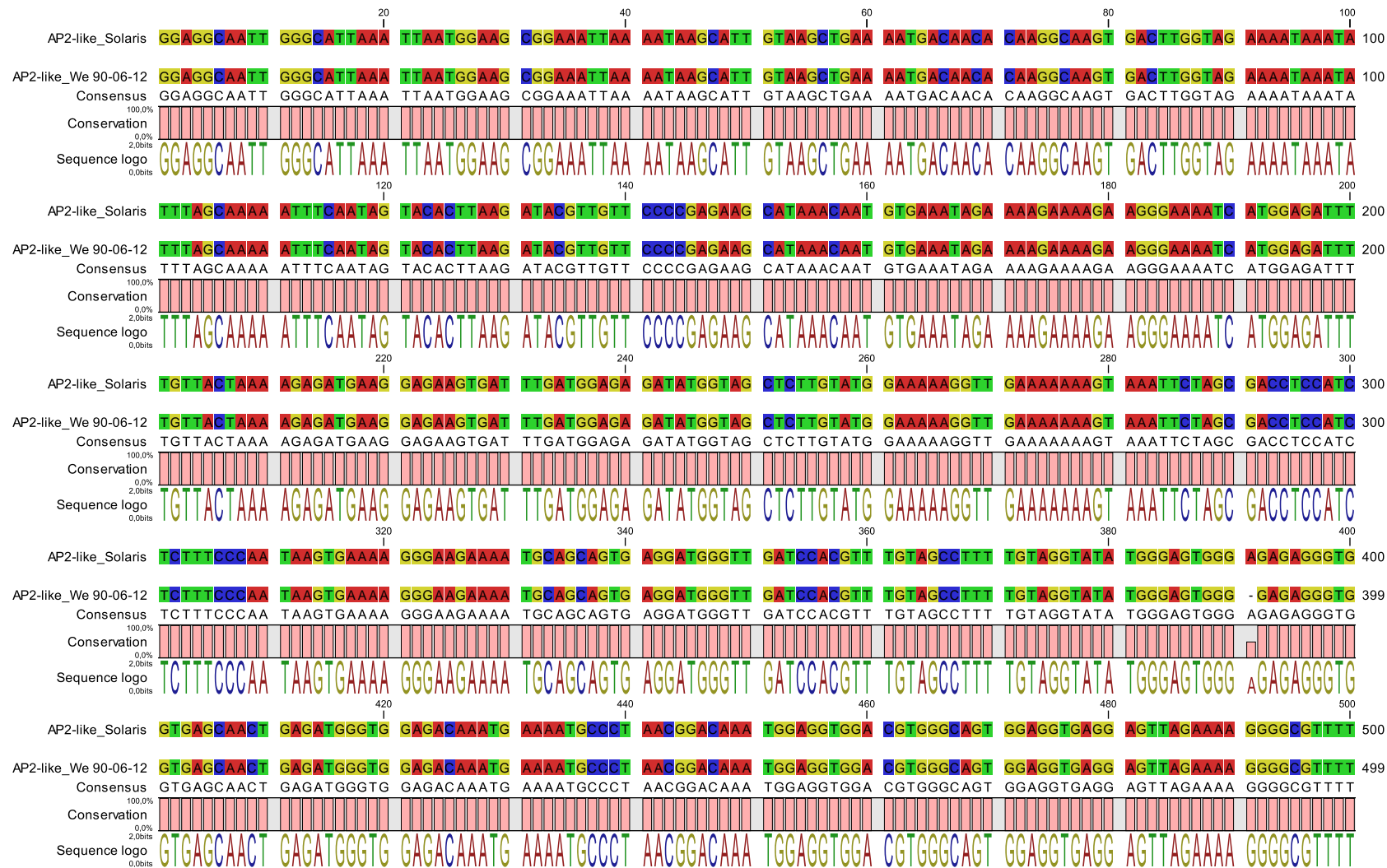

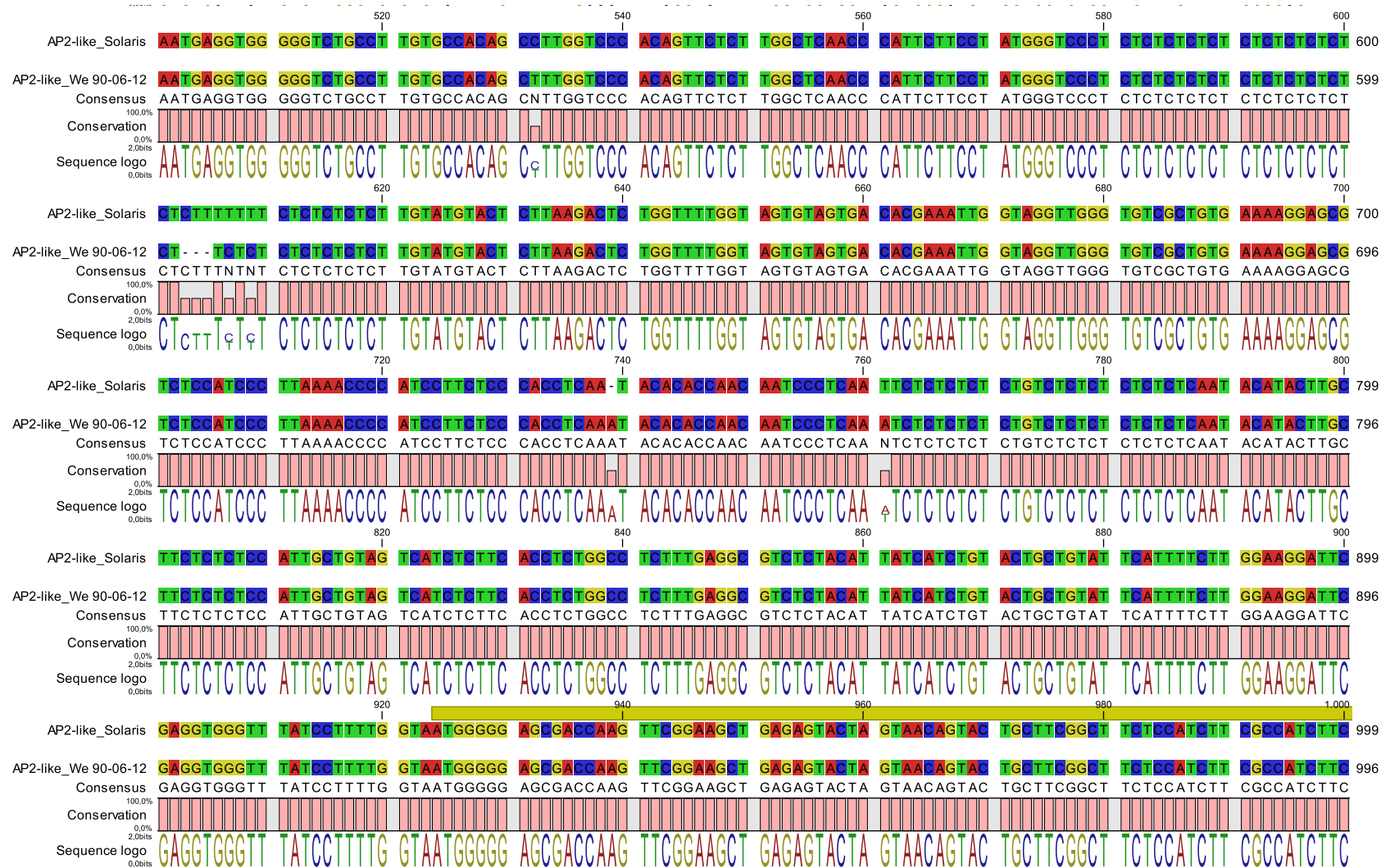

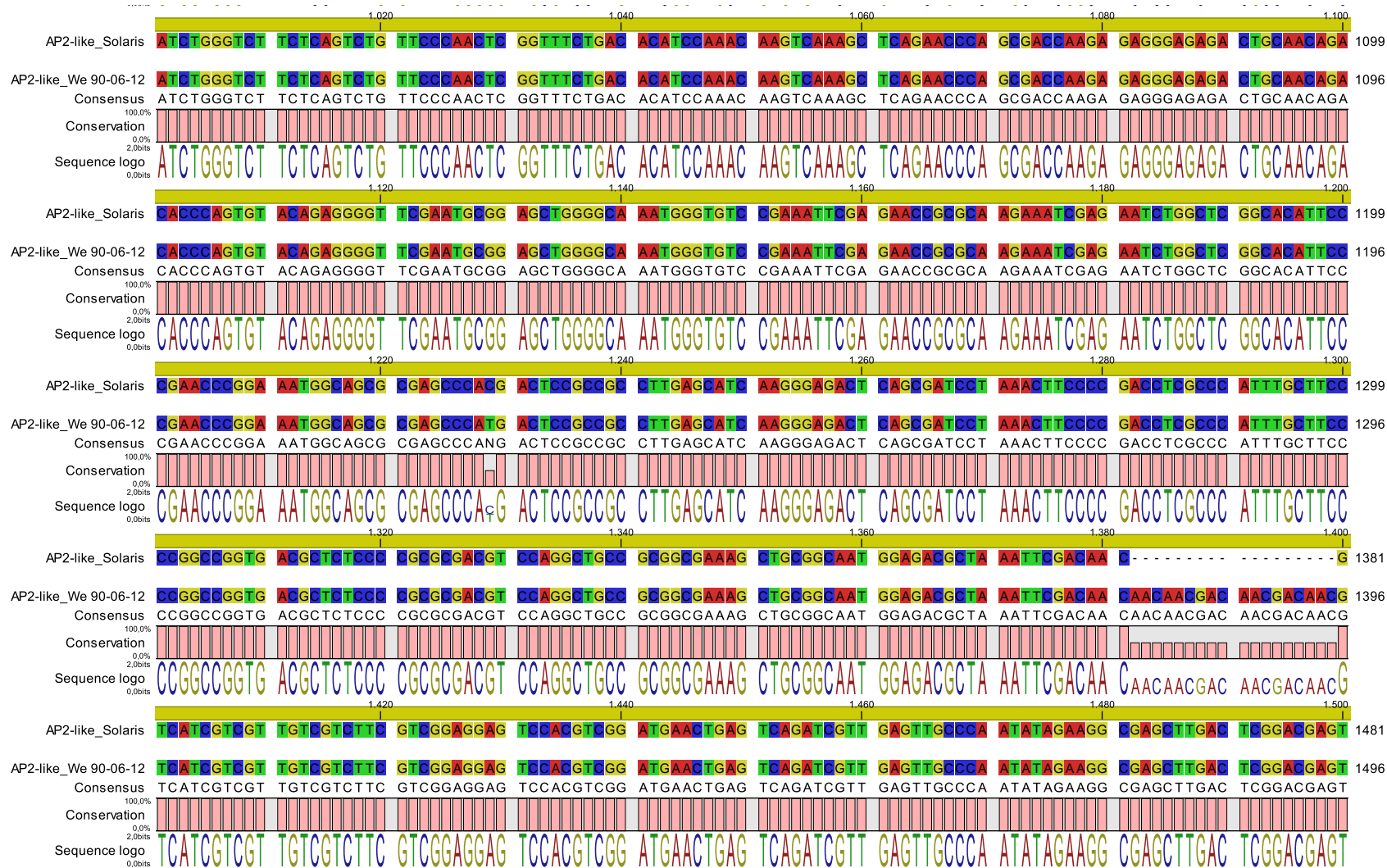

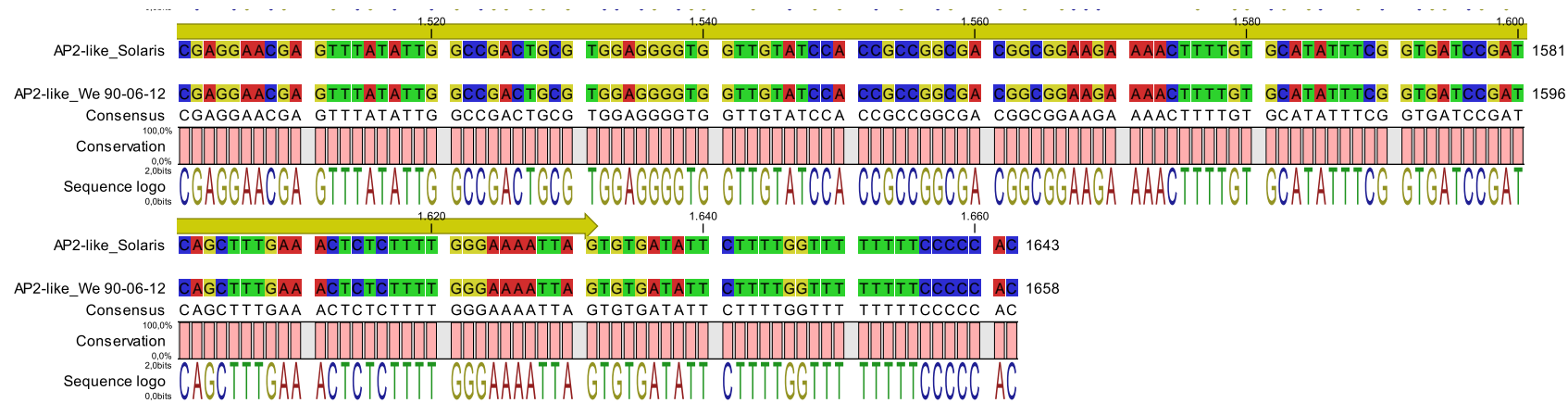

Supplement: Supplementary file 1 [file plants-13-02624-s001.zip › Figure S4_Final_SOL-WE90_AP2.pdf]
